# Supplementary material for: The Bivalent Bromodomain Inhibitor MT-1 Inhibits Prostate Cancer Growth
Source: Cancers (Basel). 2023 Jul 28;15(15):3851. doi: 10.3390/cancers15153851 (PMC10416835; doi:10.3390/cancers15153851)
Supplement: Supplementary file 1 [file cancers-15-03851-s001.zip › cancers-2491010-supplementary.pdf]

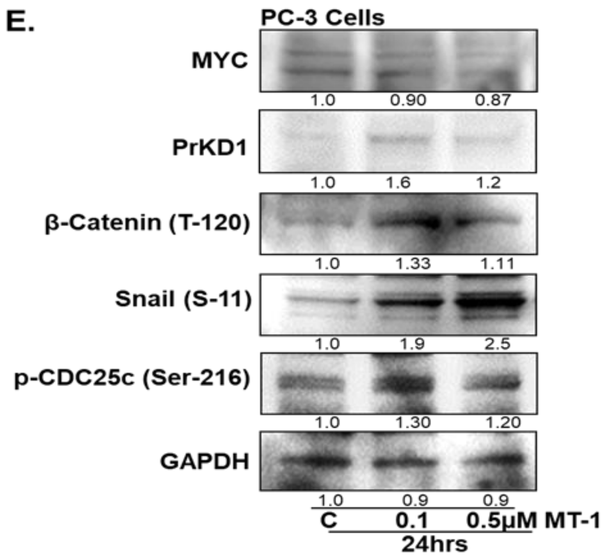

Figure 1E: Western blot analysis of PC-3 cells with MT-1 at 0.1 and 0.5μM concentrations for 24 hours reduced and increased MYC and PrKD1 protein levels respectively. Consistent with increased in PrKD expression, the known substrate phosphorylation of p-CDC25c (Ser-216), β-Catenin (T-120) and Snail (S-11) were increased from 1.11 to 2.5-fold confirming an increase in PrKD kinase activity (Fig. 1E).

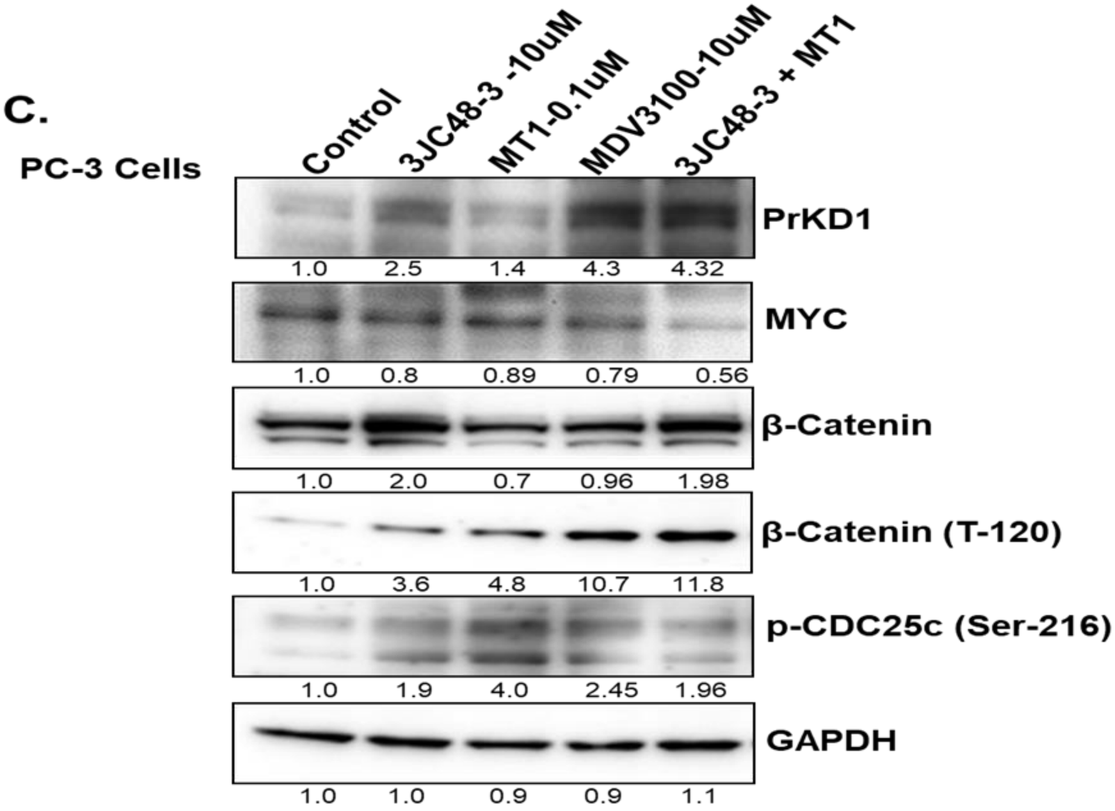

Figure 2C: Western blot (Fig 2C): Compared to control, treatment of PC-3 cells with the drugs for 24 hours reduced and increased MYC and PrKD1 protein levels respectively. Consistent with increased in PrKD1 expression, the known substrate phosphorylation of p-CDC25c (Ser-216), β-Catenin (T-120) and Snail (S-11) were increased confirming an increase in PrKD kinase activity. Like viability assay, the combination of 3JC48-3 and MT-1 was most effective compared to single drug treatments. As PrKD1 expression has been shown to be repressed by androgens), we used MDV3100, an androgen receptor antagonist, as positive control to upregulate PrKD1 expression.

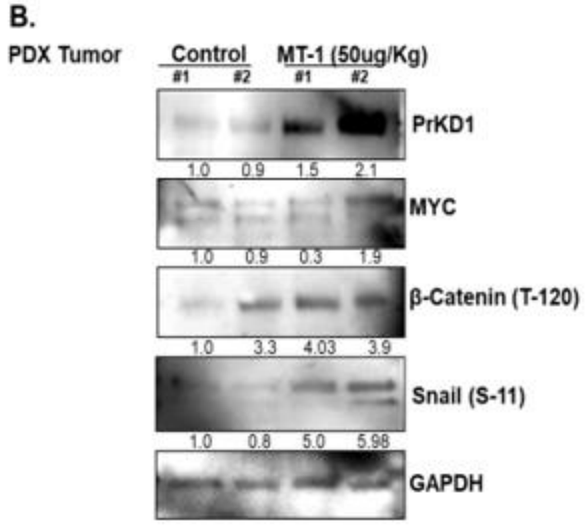

Western blot analysis of tumor samples treated with MT-1 increase expressions of PrKD1 along with increased substrate phosphorylation of Snail (S-11) and β-Catenin (T-120). GAPDH is loading control for protein (Fig 4B).
